# Spring Diseases

**Published:** 1860-05

**Authors:** 


### SPRING DISEASES.

ANY housekeeper would be considered demented who would keep up as fierce a fire on the hearth in the spring as in midwinter. On the contrary as the days grow warmer, less and less fuel is used, until the fire is not kindled at all. One of the two main objects of eating is to keep the body warm; and it need not be argued that less warmth is required in summer than in winter; but if we eat as heartily as the spring advances as we did in cold weather, we will burn up with fever, because we have made too much heat. The instincts of our nature are perfectly wonderful. To our shame is it, that we not only do not heed them, but oppose them, fight against them with an amazing fatuity. As the warm weather comes on, we are all conscious of a diminution of appetite, and we either begin to apprehend we are about to get sick, or set about stimulating ourselves with tonics, and bitters, and various kinds of teas, with a view to purifying the blood. How many swills of sassafras-tea has the reader taken to that end! No such purification would be needed, if we would follow nature's instincts, and eat only with the inclination she gives us, instead of taking tonics to make us eat more, when we actually require less.

Observant persons have noticed that as spring comes on, there is less relish for meats of all kinds, and we yearn for the early spring vegetables, the "greens," the salads, the spinnage, the radishes, and the like. Why? Just look at it! Meats have more than fifty per cent of carbon, of the heat-forming principle. Vegetables and berries have ten per cent, five per cent, one per cent of heat! Potatoes have eleven per cent, turnips three per cent, gooseberries only one.

Literally, incalculable are the good results which would follow a practical attention to these facts. Those who are wise will take no tonics for the spring, will swallow no teas to purify the blood, nor imagine themselves to be about getting sick, because they have not in May as vigorous an appetite as in December, but will at once yield themselves to the guidance of the instincts, and eat not an atom more than they have an inclination for to the end of a joyous spring-time and a summer of glorious health; while those who will eat, who will stimulate the stomach with tonics, and "force" their food, must suffer with drowsiness, depression and distressing lassitude ; and while all nature is waking up to gladness and newness of life, they will have no renovation and no well-springs of joyous and exuberant health.

